# Radiological assessment and surgical management of cervical spine involvement in patients with rheumatoid arthritis

**DOI:** 10.1007/s00296-022-05239-5

**Published:** 2022-11-15

**Authors:** Timoleon Siempis, Charalampos Tsakiris, Zikou Anastasia, George A. Alexiou, Spyridon Voulgaris, Maria I. Argyropoulou

**Affiliations:** 1 Department of Neurosurgery, Medical School, University of Ioannina, School of Medicine, Ioannina, Greece; 2 Department of Radiology, Medical School, University of Ioannina, Ioannina, Greece

**Keywords:** Rheumatoid arthritis, Cervical vertebrae, Antirheumatic agents, Diagnostic imaging, Radiography, Prevalence, Surgical procedure, Post-operative complication, Joint instability, Spine

## Abstract

The purpose of the present systematic review was to describe the diagnostic evaluation of rheumatoid arthritis in the cervical spine to provide a better understanding of the indications and options of surgical intervention. We performed a literature review of Pub-med, Embase, and Scopus database. Upon implementing specific inclusion and exclusion criteria, all eligible articles were identified. A total of 1878 patients with Rheumatoid Arthritis (RA) were evaluated for cervical spine involvement with plain radiographs. Atlantoaxial subluxation (AAS) ranged from 16.4 to 95.7% in plain radiographs while sub-axial subluxation ranged from 10 to 43.6% of cases. Anterior atlantodental interval (AADI) was found to between 2.5 mm and 4.61 mm in neutral and flexion position respectively, while Posterior Atlantodental Interval (PADI) was between 20.4 and 24.92 mm**.** 660 patients with RA had undergone an MRI. A pannus diagnosis ranged from 13.33 to 85.36% while spinal cord compression was reported in 0–13% of cases. When it comes to surgical outcomes, Atlanto-axial joint (AAJ) fusion success rates ranged from 45.16 to 100% of cases. Furthermore, the incidence of postoperative subluxation ranged from 0 to 77.7%. With regards to AADI it is evident that its value decreased in all studies. Furthermore, an improvement in Ranawat classification was variable between studies with a report improvement frequency by at least one class ranging from 0 to 54.5%. In conclusion, through careful radiographic and clinical evaluation, cervical spine involvement in patients with RA can be detected. Surgery is a valuable option for these patients and can lead to improvement in their symptoms.

## Introduction

Rheumatoid arthritis is estimated to affect 0.67% of the world population [1]. This systematic inflammatory process leads to synovitis and pannus formation which primarily involves peripheral joints. However, the inflammatory sequelae of RA can also involve the cervical spine region causing odontoid erosion, loosening of the cervical ligaments and subsequent spinal dislocation. This process was reported in 26.65% of cases in a large cohort of patients diagnosed with RA [2]. Interestingly, corticosteroid use, hands and feet involvement as well as high BMI were proved to be strong predictors of cervical involvement in patients diagnosed with RA [3].

The most common complication of the inflammatory cascade that affects the cervical region is atlantoaxial subluxation (AAS) with instability between C1 and C2 vertebrae. AAS can be divided anatomically to anterior, posterior and vertical AAS. The latter can cause superior migration of the odontoid into the foramen magnum resulting in a condition which is called cranial settling (CS) [4].

Sub-axial subluxation (SAS) is diagnosed when a vertebrae moves forward by at least 3 mm toward the lower adjacent vertebra and it mainly affect C3 to C7. In more severe cases, this process may result in spinal cord compression [5]. Cervical spine involvement in RA can manifest as persistent headaches or a positive Lhermitte’s sign. This sign is the result of compression of the greater and lesser occipital nerve and is usually described as a shooting electrical sensation running down the back when the neck is flexed [6]. Early detection of cervical spine instability is crucial as it can lead to significant neurological morbidity and worsening of quality of life due the compression of critical neural structures [7].

Radiological assessment has an important role to play in the evaluation of cervical spine involvement in RA. According to the European League Against Rheumatism (EULAR), a lateral radiography should be obtained both in neutral and flexion position [8]. The atlantoaxial joint is best assessed using the anterior atlanto-dental interval (AADI) and the posterior atlanto-dental interval (PADI) [9]. Conventional Radiography (CR) does not provide good information regarding synovial inflammation or soft tissue structural changes. For this reason, the leading imaging technique for the assessment of cervical spine involvement in patients with rheumatoid arthritis is MRI. This imaging method enables the early detection of soft tissue involvement and spinal cord compression [10]. MRI is indicated in patients with evidence of AAS on radiographs, in those with neurological signs suggestive cervical myelopathy or radiculopathy and in patients whose symptoms are not controlled with conservative management and are candidates for surgery [11]. MRI has demonstrated greater sensitivity for the detection of synovitis, soft tissue inflammatory changes and erosions at the cranio-vertebral junction when compared to either clinical examination or conventional radiography and also plays a unique role in providing important information early in the course of RA [12]. Despite the reduction in the incidence of cervical spine instability after the addition of the biological agents in the management of RA, surgery has still a vital role in the therapeutic approach [13, 14]. The reason this happens is that once myelopathy occurs, further neurological progression cannot be prevented with neither DMARDS nor biological agents [15]. In a study of patients with RA and myelopathy who did not wish to have surgery, 76% had further neurological deterioration, and all patients were bedridden within 3 years of development of myelopathy [11].

The ultimate goal of surgery is to relieve any neurologic compression and reduce any instability therefore preventing any further neurologic deficit. Many different surgical techniques have been developed over the past years. Occipito-cervical fusion is indicated in cases of cranial settling and C1–C2 fusion is the procedure of choice for atlantoaxial subluxation [16].

In this review, we will discuss the value of CR and MRI evaluated spine disease in RA and provide an overview of the modern surgical techniques as well as outcomes.

## Methods

**Fig. 1:**
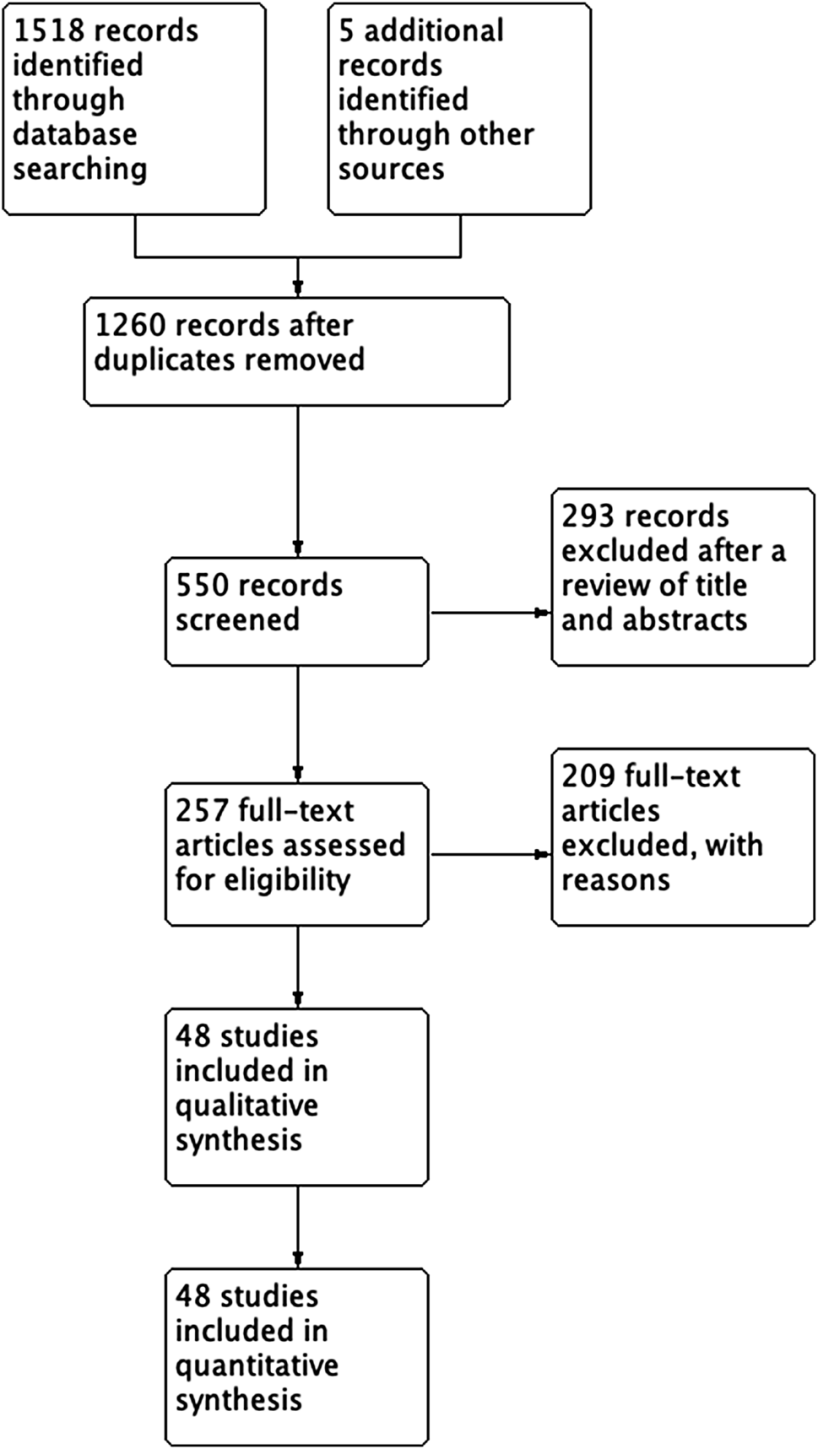
Flow chart diagram presenting the selection of eligible studies

We performed a literature review on Pub-med/Medline, Embase, and Scopus database and assessed all relative references for eligible studies. The following search algorithm was used: “arthritis, rheumatoid” [MeSH Terms] OR (“arthritis” [All Fields] AND “rheumatoid” [All Fields]) OR “rheumatoid arthritis” [All Fields] OR (“rheumatoid” [All Fields] AND “arthritis” [All Fields])) AND (“cervical vertebrae” [MeSH Terms] OR (“cervical” [All Fields] AND “vertebrae” [All Fields]) OR “cervical vertebrae” [All Fields] OR (“cervical” [All Fields] AND “spine” [All Fields]) OR “cervical spine” [All Fields]) AND atlantoaxial [All Fields]. All studies between January 1983 and June 2020 were reviewed. This yielded a total of 259 results. Each of the results was independently reviewed by two authors T.S and C.T. Furthermore, the relative reference lists were manually searched to find eligible studies. The inclusion criteria were: (1) Studies including RA patients with CS available CR or MRI imaging (2) Studies that included patients undergoing surgery for cervical spine instability attributed to RA and it was clearly stated that they were followed post-operatively, (3) Studies including symptomatic patients with RA with regards to cervical involvement. We excluded: (1) Case reports, case series, review of literature, (2) Non-English literature. Improvement in Ranawat scale was considered when there was an improvement of at least 1 Ranawat class above the preoperative assessment. The postoperative outcome was evaluated at the end of the reported follow-up period rather than immediately after surgery. The detailed study flow chart is presented in Fig. 1.

The results were then categorized according to their theme as follows:

- Commonly reported findings in plain radiographs of patients with RA as well as the incidence of Atlantoaxial and Sub-axial subluxation
- Evaluation of the outcomes of cervical spine surgery in patients with RA
- The deterioration of Sub-axial instability preoperatively

The articles selected were then classified according to an evidence-based classification proposed by Wright et al. [17]:

I. High-quality randomized controlled trial
II. Lesser quality randomized controlled trial; prospective comparative study
III. Case–control study; retrospective comparative study
IV. Case series. Expert opinion.

## Radiological findings of CS in patients with RA using plain radiography

Plain radiographs are valuable tool for screening for cervical spine involvement (CS) in patients with RA. The main radiological findings in plain radiographs are described below.

AAI is a condition that affects the stability of the atlanto-axial joint and has been associated with laxity or rupture of the transverse ligament and erosions or fracture of the odontoid process. AAI accounts for approximately 65% of the total subluxations of the spine [18, 19]. Measurement of the anterior atlanto-dental interval (AADI) and posterior atlanto-dental interval (PADI) is used to evaluate the AAI. AADI is defined as the interosseous distance between posterior surface of C1 and the anterior surface of C2. The normal value of AADI in adults should be less than 3 mm. In a study evaluating AADI and PADI during motion of cervical spine using lateral fluoroscopy, AADI in neutral position measures 1.62 mm ± 0.62, whereas at maximal flexion and extension, it increased to 1.88 mm ± 0.85 and 1.63 mm ± 0.61 respectively [20, 21].

AADI > 5 mm is an indicator of clinically significant AAS instability. While AADI > 8 mm has been proposed as the optimal cut-off for surgical management, the various cut-offs range from 6 to 10 mm [5]. It is important to note that the reliability of AADI as an indicator of atlantoaxial instability is limited in patients with cranial settling. In this case, AADI might be mistakenly regarded as decreased when in fact the patient suffers from severe instability [22].

Posterior atlanto-dental interval (PADI) is defined as the distance between the dens and the posterior arch of C1. This distance reflects the width of the spinal cord on C1–C2 level and typically measures between 19 and 27 mm [23]. It is a reliable predictor of the width of the atlantoaxial canal and has shown to be a reliable indicator of myelopathy. This diagnostic tool can also help the surgeon predict postoperative neurologic outcome [24]. PADI < 14 mm has been correlated with adverse surgical outcomes and is more commonly complicated with vertical subluxation [25, 26].

### Sub-axial subluxation in CS in RA patients

Sub-axial subluxation (SAS) is diagnosed when the vertebra is horizontally displaced by at least 3.5 mm forward to the adjacent vertebra in a lateral radiograph. This instability involves the lower joints of the cervical spine (C3–C7). The prevalence of SAS in patients who have not undergone surgery in the cervical spine ranges from 15 to 22% [27]. SAS is sometimes described according to the spinal canal diameter. Clinically significant stenosis occurs when the width of the sub-axial canal is less than 14 mm [28]. The patients with sub-axial cervical spine stenosis are more likely to be classified as a Ranawat class II or III (*P* = 0.01; odds ratio (OR) = 11.43] [29].

### Basilar invagination in CS in RA patients

Basilar Invagination (BI), previously described as cranial settling (CS), is caused by the superior elevation of the odontoid into the already limited space of foramen magnum. The degree of dens displacement was first described with Chamberlain (DOCL) and McGregor line (DOMG). Modern diagnostic criteria include Ranawat criterion, Redlund-Johnell criterion and Clark station. Modified Ranawat measurement extends from midpoint of base of C2 to level of C1, along long axis of odontoid [30]. Redlund-Johnell criterion is the minimum distance between the midpoint of the base of C2 and McGregor’s line [31, 32]. In the Clark station, the dens is divided into three equal parts and determined by the level at which the anterior arch of C1 falls.

Because none of aforementioned criteria individually results in satisfactory sensitivity and specificity, Riew et al. suggested that the presence of CS is best evaluated using a combination of the Clark station, Ranawat criterion, and the Redlund-Johnell criterion. If any of the three criteria is positive, there is a strong probability of cranial settling (sensitivity of 94%, negative predictive value of 91%). However, this method yields only a positive predictive value of 56%, thus making the use of CT or MRI mandatory [33].

Basilar invagination can result in root compression. This complication can be predicted with the use of cervical–medullary angle, which is defined as the angle between a line drawn along the anterior aspects of the cervical–medullary cord and another line along the medulla. The normal angle is 135°–175°. Patients with a cervical–medullary angle < 135° may suffer from cranial settling and clinical signs of C2 root pain [34]. Plain radiographs have not been found to be a reliable diagnostic imaging modality for the detection of BI. Chung et al. reported that Vertical Subluxation (VS) was identified with plain radiographs in 2.30% of patients, whereas in 33.5% MRI scans [35].

## Radiological findings of CS in patients with RA using MRI

MRI is mainly used to assess the presence of synovitis, bone marrow edema (BME), odontoid erosions, anterior, posterior or superior VS, and alterations of the cervico-medullary angle.

### BME in CS in RA patients

Bone marrow edema (BME) is defined as a poorly defined area of low signal within bone on T1-weighted images that shows high signal intensity on Short Tau Inversion Recovery (STIR) images. The degree of BME is quantified according to an MRI scoring system by measuring the extension of BME in the dens axis as well as in the corpus, facet joints, and spinous process of C2–C7: the dens axis is scored for BME with a scoring of 0 when there is no BME, 1 when BME covers < 1/3 of the bone surface and 2 when BME covers ≥ 1/3 of the bone surface [36]. The presence of BME is strongly associated with the degree of synovitis and it has been proved to be a strong predictor of progressive erosion. In terms of its clinical value, the degree of BME found in MRI has not been proved to be associated with the degree of neck pain [37, 38].

### Synovitis in CS in RA patients

Synovitis, as proposed by the OMERACT group, is defined as a thickening of the synovial membrane at the atlantoaxial joint (C1–C2), showing increased water content in fat-suppressed T2-weighted and STIR sequences or an abnormal post-gadolinium enhancement on T1-weighted images [39].

Recent research has focused on the quantitative assessment of inflammatory activity with the introduction of biomarkers such as synovial volume and synovial perfusion with the use of dynamic contrast-enhanced MRI (DCE-MRI) [40]. Fasler et al. demonstrated that DCE-MRI was feasible in 80% of patients despite potential artifacts attributed to the anatomy of the cervical region. Using this imaging modality, pannus volume was quantified and calculated at 5.7 ± 2.4 mL [41]. This kind of quantitative MRI techniques is crucial, since the differentiation between normal enhancement and enhancement attributed to synovitis, especially early in the course of RA, remains a challenge up to date [42].

One of the main benefits of MRI is that it enables early detection of pannus tissue, even when radiographic findings are negative and thus early identification of ligament destructions [43]. Many studies have correlated early atlantoaxial involvement with a number of prognostic factors, the most important of which are anti-citrullinated protein antibodies (ACPAs), high disease activity, as well peripheral erosive disease [36]. With regard to cervical spine, STIR images alone have been shown to be as sensitive and reliable in terms of detection of inflammation as T1-weighted post-contrast images. Omitting the administration of contrast agent saves time and is associated with less cost [44].

### Erosion of the odontoid process in CS in RA patients

Odonotoid erosion is a common radiographic finding in patients with RA with a prevalence of 43–47% [43]. Erosion of the odontoid process is defined as a bone defect with sharp margins, visible in two planes. The degree of erosion can be quantified by a scale from 0 to 5 or being dichotomized to whether erosion is present or absent according to the simple erosion narrowing score (SENS) method [45]. Odontoid erosion further deteriorates atlantoaxial instability as it is highly associated with posterior AAS [19]. Furthermore, the chance of neurological dysfunction increases by 5 times when erosion is present [46].

### Stenosis of the cervical spinal cord in RA patients

Upper cervical cord or brainstem compression occurs when the subarachnoid space is obstructed which is evident when the cerebrospinal fluid disappears in both the anterior and posterior subarachnoid spaces on T2-weighted images. A deformity of the spinal cord or brainstem appears on MRI imaging as decreased cord diameter at the level of subarachnoid space obstruction compared with the cord diameter superior or inferior to the stenotic level. Sub-axial spinal cord compression is present in cases of obstruction of the subarachnoid space and deformity of the medulla.

Stenosis of the spinal canal is primarily found in the sub-axial level (85%) followed by the atlantoaxial level (44%). Interestingly, a spinal stenosis detected with an MRI has not been found to be correlated with a clinically evident neurological dysfunction except for a stenosis that occurs in the sub-axial level [29].

Despite the significance of MRI in the early detection of spinal cord compression, its additional value compared to function plain radiographs in diagnosing spinal cord compression has been doubted [47]. According to a recent randomized clinical trial, patients who present with symptoms of myelopathy from an acutely inflamed atlantoaxial joint can be relieved by intraarticular injection of steroids [48].

New imaging techniques are gradually introduced in clinical practice and enable the clinician to diagnose abnormalities of the spinal cord before the onset of irreversible damage. One of them is DWI which has been found to be useful in the identification of spinal cord compression due to anterior AAS by measuring the ADC values at C1 level (ADC_1_). The latter have been found to be higher in the group with anterior AAS than in the group without AAS (*P* < 0.001) [49].

## Studies looking at cervical involvement in patients with RA with plain radiographs and MRI

We identified 18 studies evaluating cervical spine involvement in patients with rheumatoid arthritis with plain radiographs that met our inclusion and exclusion criteria.

A total of 1878 patients were included (Table 1). Useful views for evaluation are upright AP and lateral, open-mouth (odontoid view), and flexion–extension for detection of instability. AAS was the most frequently encountered cervical abnormality, with its frequency ranging from 16.4 to 95.7% in plain radiographs. SAS diagnosis was found in 10–43.6% of cases. ADI was found to be ranging from 2.5 to 4.61 mm in neutral position. PADI ranged from 20.4 to 24.92 mm.

**Table 1:** Summary of all the studies reporting the incidence of AAS and SAS

References	Patients (number)	Disease duration (years)	AAS (No of patients)	SAS (No of patients)	Mean AADI (mm)	Mean PADI (mm)
Cardoso et.al. [50]	35	15	15	7	3.4	NA
Alcala et.al. [51]	80	11	12	13	4.3	20.4
Younes et.al. [52]	40	10	9	4	4	23.5
Zikou et.al. [53]	165	12.4	34	72	2.5	NA
Chung et.al. [35]	242	NA	117	33	4.61	24.92
Macovei et.al. [54]	20	NA	13	7	NA	NA
Hagenow et.al. [55]	21	9	21	NA	NA	NA
Kanayama et.al. [56]	47	11	NA	NA	4.5	NA
Taniguchi et.al. [57]	30	12.1	8	NA	1.8	NA
Maeda et.al. [58]	24	NA	18	NA	NA	NA
Blom et.al. [59]	134	9	22	NA	NA	NA
Yurubu et.al. [60]	228	NA	57	7	NA	NA
Yurubu et.al. [61]	267	NA	85	74	NA	NA
Yan et.al. [62]	71	18.2	68	NA	NA	NA
Takahasi et.al. [63]	220	11.1	79	28	NA	NA
Neva et.al. [27]	154	NA	27	24	NA	NA
Imagama et.al. [25]	100	13	45	23	NA	NA

With regard to the evaluation of CS in RA patients with MRI, 13 studies were found. The most commonly reported MRI findings in patients with rheumatoid arthritis are found in Table 2. In a total of 660 patients, AAS and SAS frequency ranged from 4 to 87.8% and 0 to 13.6% respectively. Odontoid erosion was a frequently encountered finding reported in 16–85.36%. A pannus diagnosis ranged from 13.33 to 85.36%, while evidence of spinal cord compression was reported in 0–13% of cases. What is more, very few studies reported BME either at the atlantoaxial or sub-axial level.

**Table 2:** Most commonly reported MRI findings

References	Patients (number)	Mean duration (years)	SCC (No of patients)	Angle < 135 (No of patients	BME Odontoid (No of patients)	Subaxial (No of patients)	Pannus (No of patients)	AAS (No of patients)	SAS No of patients)	Odontoid Erosion (No of patients)
Baraliakos et.al. [38]	34	13.2	0	N/A	4	17	N/A	N/A	N/A	N/A
Carotti et.al. [36]	50	< 1 year	1	2	6	2	12	2	0	8
Chung et.al [35]	242	NA	46	N/A	N/A	N/A	61	117	33	107
Fasler et al. [41]	10	NA	N/A	N/A	N/A	N/A	N/A	N/A	N/A	N/A
Jeromel et al. [64]	27	< 2 years	N/A	N/A	N/A	N/A	N/A	N/A	N/A	N/A
Magerelli et al. [65]	20	< 1 year	N/A	N/A	N/A	N/A	5	N/A	N/A	N/A
Narvaez et al. [66]	41	14	24	4	N/A	N/A	35	36	N/A	35
Olah et.al. [67]	49	9	N/A	N/A	N/A	N/A	12	13	N/A	8
Suppiah et al. [12]	30	15.5	N/A	N/A	9	10	N/A	N/A	N/A	N/A
Zikou et al. [68]	51	12.4	3	3	N/A	N/A	44	7	5	12
Younes et al. [52]	40	10	1	1	N/A	N/A	25	12	4	27
M Schwarz-Eywill et al. [69]	36	NA	10	N/A	N/A	N/A	7	25	N/A	N/A
Daoud et al. [70]	30	NA	1	N/A	N/A	N/A	4	5	1	2

## Surgical management of CS involvement in RA patients

Surgical management is mainly indicated when the patient suffers from myelopathy and/or neurological dysfunction. Surgery typically consists of a decompressive procedure to relieve the compression of the spinal cord in combination with a fusion procedure which aim to eradicate any instability. This stabilization process also prevents further progression into VS or SAS.

Atlantoaxial stabilization can be performed via either an anterior or posterior approach depending on the site of compression and the surgeon’s preference [71]. Sunahara et al. demonstrated the vital role of cervical spine surgery in a sample of 21 patients with RA who were not managed surgically. 16 of the patients deteriorated at follow-up and the chance of surviving 7 years after the onset of myelopathy was 0% [72]. Surgery has also played a role in pannus regression, an inflammatory complication of RA. Bydon et.al demonstrated that the mean volume of pannus decreased by 44% in patients managed with posterior fusion with or without decompression [73].

The most commonly employed technique for C1–C2 instability is C1–C2 fusion. This technique is mainly indicated in patients without other coexisting cervical deformities. With regard to preoperative considerations, methotrexate should not be discontinued. As for the biological agents, it is recommended that they should be stopped one week prior to the operation although this view has been challenged by Kawakami et.al who found no significant adverse effects postoperatively in patients who continued taking biological agent before surgery. On the other hand, Ozen et al., in a large series of 11.623 patients found that the incidence of surgical infection was higher in patients taking tumor necrosis factor α inhibitors (TNFis) when compared to the ones taking conventional DMARDS [74, 75].

The technique most commonly employed is the one introduced by Harms and Melcher and involves the fixation of posterior C1 lateral mass and C2 pedicle using screws and rods. In this procedure, the surgeon must seriously consider the course of the vertebral artery (VA), as the risk of injuring the latter is estimated at 4.1%. Patients with RA may have a special anatomy of the cervical region as they were found to have smaller left internal height 4.21 ± 1.63 and a pedicle width of 4.11 ± 1.05 of C2 [76–78].

Furthermore, after the comparison between anterior and posterior approach, Chieng found in a meta-analysis that 68% of patients treated with an anterior approach improved their neurological status, while the same figure for patients managed with posterior approach was 98% [79]. When AAI occurs posteriorly, occipito-cervical fixation is mandatory. The latter is also indicated as a reoperation technique when the instrumentation in C1–C2 fail. This occurred in a series of 66 patients in 11% of cases at a mean time of 28 months. The most important consideration in the occipito-cervical fusion is the occipito-cervical angle, as it has a significant impact on the postoperative outcome. Two cervical angles that have proved of great importance are Occipitoaxial Angle and Posterior Occipitocervical Angle which normally measure 14.5° ± 3.7° and 108.2° ± 8.1°, respectively. The latter has been correlated with the need for reoperation when elevated [80, 81].

With the regard to the outcome of cervical spine approaches used to treat myelopathy, this is evaluated with the use of Japanese Orthopaedic Association scoring system (JOA). The latter is a questionnaire which is answered by the patients themselves and assesses preoperative versus postoperative neurological status. JOA score is significantly improved postoperatively in patients without SAS. To overcome some limitations of JOA score, a new score was introduced called the Japanese Orthopaedic Association Cervical Myelopathy Evaluation Questionnaire (JOACMEQ), which focuses more on the patients’ quality of life and general health [82]. Preoperative Ranawat classification is also an important predictor of postoperative outcome. Postoperative improvement is defined as improvement of at least 1 Ranawat classification above the preoperative assessment. Interestingly, outcome of surgery in terms of neurological function is not affected by the degree of dislocation preoperatively [83]. The degree of atlantoaxial fusion can be evaluated by comparing preoperative ADI versus postoperative ADI. The visual analog scale (VAS) is frequently used to measure treatment outcome in patients with cervical spine disorders in a scale of 0–100 mm [84].

### Endoscopical approach

An anterior approach is mainly indicated when there is anterior spinal cord compression due to irreducible basilar invagination (BI) and/or rheumatoid pannus (RP). Otherwise, posterior C1–C2 fusion is the preferred technique. The former approach enables direct decompression, while the latter enables stabilization of the alignment of the cervical spine. The optimal method to treat cervical spine instability remains still a controversy to date [85].

The odontoid can be accessed either with the trans-oral or the endo-nasal approach. The latter, which is often referred to as Endoscopic Endo-nasal Approach (EEA) provides a better surgical field and presents less postoperative complications as opposed to the trans-oral approach (TOA), but no clear superiority of the EEA over the endoscopic TOA has been proved [86, 87]. This procedure is in most cases accompanied by posterior fusion to prevent subsequent C1–C2 instability. However, Iacongeli et al. presented a case series of 7 patients which showed that C1 ach can be preserved. The main advantage of this less invasive approach is that it prevents future cranial settling and progression of C1–C2 instability [88]. Despite its crucial benefits, it is important to note that trans-nasal approach has been more frequently associated with intra-operative CSF leaks than the trans-oral approach (30 and 0.3% respectively). This leak, however, remains in the follow-up period only in a minority of patients. In a review of literature, Morales Varelo et al. included 72 patients undergoing EEA due to various etiologies including 28 with rheumatoid pannus. This study showed that EEA is a safe approach with a procedure-related mortality of just 1.4% [89].

Odontoidectomy can also be performed via the trans-cervical root which offers significant improvement in terms of myelopathy and is accompanied by only few complications, such as a urinary tract infection, upper airway swelling, and dysphagia. This is the technique of choice once cranial settling has occurred [90].

Novel surgical techniques are gradually introduced in clinical setting. For instance, cervical pedicle placement with intraoperative CT guidance in 20 consecutive patients with rheumatoid arthritis was reported to have an overall malposition rate of 2.4%. Jha et al. proposed an alternative technique of C1–C2 posterior fusion with use of operative video, thus minimizing intra-operative blood loss and C2 dorsal root ganglia [91]. It is worth mentioning that in the event of surgical failure, a conservative approach has been proved to reduce rheumatoid pannus considerably using a rigid cervical collar [92].

## Studies looking at the surgical management of CS in RA patients

Looking at the studies assessing surgical management of CS involvement in patients with RA, we found a total of 17 studies which assessed postoperative outcomes and seven studies that evaluated the potential rise in the incidence of postoperative subluxation compared to the preoperative ones. There were studies that evaluated both outcomes and were therefore reported twice in each individual table.

In Table 3, we present the surgical outcomes of the included studies. This was evaluated by the degree of increase in JOA score, whether patients were classified at lower Ranawat class after surgery, by the proportion of patients which AAJ fusion was successful and by the drop in mean AADI postoperatively. JOA score increased postoperatively in all but one study (Kurogochi et.al) in which it remained stable. AAJ fusion was successful in the vast majority of patients undergoing cervical stabilization surgery and more specifically ranged from 45.16 to 100% of cases. With regard to AADI, it is evident that its value decreased post-operatively in all studies. Furthermore, an improvement in Ranawat classification was variable between studies with a reported improvement frequency by at least one class ranging from 0 to 54.5%.

**Table 3:** Summary of postoperative outcomes

References	Procedure	Patients (No)	JOA (pre)	JOA(post)	Ranawat improvement	AAJ fusion (No of patients)	AADI pre (mm)	AADI post (mm)
Bydon et. al. [73]	C1–C2 fixation	30	NA	NA	7	NA	NA	NA
Iacoangeli et.al. [88]	EEO	7	NA	NA	7	NA	NA	NA
Miyamoto et.al. [93]	C1–C2 fixation	28	11.1	14.4	NA	NA	NA	NA
Park et.al. [94]	C1–C2 fixation	24	NA	NA	NA	NA	NA	NA
Sorimachi et.al. [95]	C1–C2 fixation	31	NA	NA	NA	14	NA	NA
Uei et.al. [96]	C1–C2 fixation	33	11.37	16.64	18	NA	7.93	2.41
Vanek et.al. [97]	C1–C2 fixation	29	NA	NA	NA	25	8.7	0.7
Janssen et.al. [98]	C1–C2 fixation	9	12	14.6	NA	NA	NA	NA
Ryu et.al. [99]	C1–C2 fixation	33	15	16	NA	32	7.6	2.5
Ryu et.al. [99]	C1–C2 fixation	25	15	16	NA	25	8	2.3
Kurogochi et.al. [100]	C0–C1 or C1–C2 fusion	6	11.2	11	0	NA	NA	NA
Bhatia et.al. [101]	C1–C2 fixation	66	NA	NA	NA	NA	NA	NA
Bhatia et.al. [101]	OCF	61	NA	NA	NA	NA	NA	NA
Clarke et.al. [102]	C1–C2 fixation	33	NA	NA	NA	29	NA	NA
Werle et.al. [103]	C1–C2 fixation	46	NA	NA	NA	45	NA	NA
Yoshida et.al. [104]	C1–C2 fixation	34	NA	NA	NA	NA	7.29	2.64
Nagaria et.al. [105]	C1–C2 fixation	36	NA	NA	12	35	5.8	2.4

In Table 4, we present the studies evaluating the incidence of preoperative sub-axial subluxation compared to the respective figure after surgery. In a sample of 213 patients undergoing posterior C1–C2 fixation postoperative subluxation ranging from 0 to 77.7%. This is in line with literature which suggests that one feared complication of posterior C1–C2 fixation is SAS.

**Table 4:** Comparison of the incidence of preoperative SAS versus Postoperative

References	Procedure	Patients	Sub-axial pre (No of patients)	Sub-axial post (No of patients)	Deterioration
Ito et.al. [106]	C1–C2 fixation	33	8	19	11/33
Yoshida et.al. [104]	C1–C2 fixation	34	0	0	0
Ishii et.al. [107]	C1–C2 fixation	58	0	19	19/58
Mukai et.al. [108]	C1–C2 fixation	28	0	5	5/28
Clarke et.al. [102]	C1–C2 fixation	33	NA	13	NA
Nagaria et.al. [105]	C1–C2 fixation	36	36	28	0
Iizuka et.al. [109]	C1–C2 fixation	25	10	12	2/25

## Discussion

RA diagnosis in CS patients is important as it is associated with high morbidity and mortality [19]. The frequency of the radiological findings of CS in RA patients varies significantly, mostly due to the study design of published articles [59]. The most commonly reported abnormality in plain radiographs of patients with RA and CS involvement was AAS followed by SAS. Interestingly, SAS was diagnosed in 10–43.6% of cases using plain radiography versus 0 to 13.6% when using MRI. MRI is a useful technique for the evaluation of soft tissue changes and bone erosive changes for instance odontoid erosion, pannus formation and spinal cord compression. RA disease duration was high in the included studies ranging from 1 to 18.1 years. It is worth noting that most of the cases with early radiological changes were noted in the MRI group of studies [36, 64, 65]. This is in line with literature that suggests radiological changes visible in plain radiography present usually at a later stage [71].

Regarding surgical management of CS in RA patients, although the aim of atlantoaxial fixation is to hinder further progression of cervical instability, post-operative SAS ranged from 0 to 77% in our sample. This complication is possibly attributed to the high mechanical stress during the surgical procedure [110]. Interestingly, Ishii et.al demonstrated that SAS occurred mainly at the C3-C4 level in 42% of cases [107].

Post-operative outcomes after cervical spine surgery in RA patients were variable. Improvement in Ranawat scale by a least one class was noted from 0 to 54% of cases. This is in line with literature which states that patients typically improve by one class after undergoing cervical spine fusion [71, 105, 111].

There have been significant changes in the management of RA during the last decades. This is mostly due to the development and approval of new biological (b) DMARDs in the treatment protocol of patients with RA. The novel target to treat (T2T) approach using conventional synthetic (cs) or targeting synthetic (ts) DMARDs, along with bDMARDs has improved significantly the outcomes and the rates of disease progression [112]. According to the NEO-RACo Study, in patients with early RA that received a triple combination of csDMARD and prednisolone, the rate of CS involvement was only 4.7% by 10 years, with AAS and SAS rate being only 1.2%. This is particularly important given the high rates of post-operative complications of surgically managed cases of RA, namely SAS. In patients with already unstable spine, surgery seems to be the mainstay of treatment to decelerate disease progression and improve long term outcomes [101].

There are limitations to this study that should be considered when interpreting the results**.** The frequency of radiological findings of CS involvement in patients with RA varies significantly. This is because the majority of the included articles are cross-sectional and retrospective and only a minority of them were designed for early RA assessment. There are multiple factors that might affect the frequency of the radiological findings in CS for instance disease duration, suboptimal treatment, seropositivity, involvement of multiple joints and the degree of joint damage. Radiological findings in cervical spine in RA patients are usually late findings. Therefore, prospective studies evaluating the incidence of cervical spine involvement in RA and the radiological abnormalities would be recommended. Furthermore, we only looked at studies including patients with recorded post-operative follow-up to assess post-operative outcomes.

## Conclusion

To the best of our knowledge, this the first study that summarizes the data regarding imaging findings and surgical management in patients with RA and cervical spine involvement. Plain radiographs have an important role to play in the evaluation of cervical spine involvement in RA. Given that radiological findings present at a later stage, screening of patient with CS involvement with CR is encouraged. MRI enables the early detection of soft tissue involvement and spinal cord compression. AAS was the most frequently encountered radiological abnormality. Although the incidence of cervical spine instability has been decreased after the addition of the biological agents in the management of RA, surgery has still a vital role in the therapeutic approach.

## Data Availability

Data available on request.

## Abbreviations

AADI: Anterior atlantodental interval
AAJ: Atlanto-axial joint
AAS: Atlantoaxial subluxation
ACPA: Anti-citrullinated protein antibodies
BI: Basilar Invagination (BI)
BME: Bone marrow edema
CR: Conventional radiography
CS: Cervical spine
CS: Cranial settling
CT: Computed tomography
DCE- MRI: Dynamic contrast-enhanced magnetic resonance imaging
DMARD: Disease modifying drugs
EEA: Endoscopic endonasal approach
JOA: Japanese Orthopaedic Association scoring system
MRI: Magnetic resonance imaging
OCF: Occipitocervical fixation
PADI: Posterior atlantodental interval
RA: Rheumatoid arthritis
SAS: Subaxial subluxation
SCC: Spinal cord compression
STIR: Short tau inversion recovery
TOA: Transoral approach
VS: Vertical subluxation
